# Dual-Function Fluorescent Probes for Neuronal Trans-Differentiation: A Promising Therapeutic Strategy in Neuroregenerative Research

**DOI:** 10.1021/acschemneuro.5c00367

**Published:** 2025-06-25

**Authors:** Pratikshya Paudel, Prabir Kumar Gharai

**Affiliations:** † Department of Chemistry, 7618 Oklahoma State University, Stillwater, Oklahoma 74078, United States; ‡ Department of Animal and Food Sciences, 7618 Oklahoma State University, Stillwater, Oklahoma 74078, United States

**Keywords:** Neurodegenerative diseases, human Mesenchymal stem cells, Neuronal trans-differentiation, Fluorescent probes, Small molecules, Neuroregenerations

## Abstract

Neurodegenerative diseases cause progressive neuronal loss, with current treatments offering only limited symptomatic relief. To overcome this, various strategies are being explored. One promising approach involves converting human mesenchymal stem cells (hMSCs) into functional neurons using a small molecule, alongside the use of fluorescent probes for real-time imaging. Therefore, developing a promising single compound that combines both differentiation-inducing and imaging capabilities without relying on growth factors could significantly advance neuroregenerative therapies and diagnostic strategies in neuroscience research.

## Graphical abstract

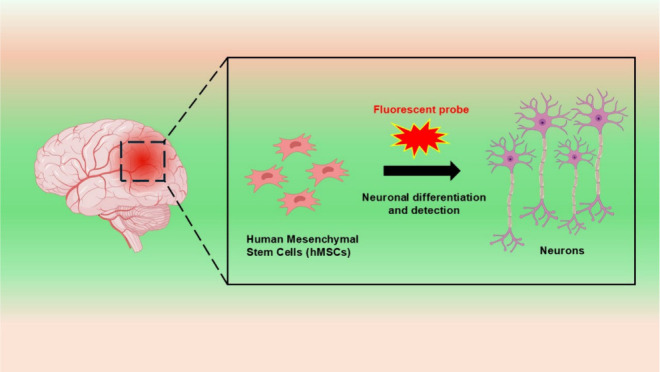

Neurodegenerative diseases, such as Alzheimer’s disease (AD), Parkinson’s disease (PD), Huntington’s disease (HD), amyotrophic lateral sclerosis (ALS), and multiple system atrophy (MSA) causes the gradual loss of neurons and their functions. These conditions lead to serious problems, such as memory impairment, motor dysfunction, and cognitive decline. Disease starts years prior to the appearance of the symptoms and the conditions get worst over time. Despite major research efforts, current treatments mainly focus on relieving symptoms or slowing disease progression for a limited time. Medications available today can only delay neuronal damage by a few months or years. As a result, there is a strong need to develop more effective treatments that can directly address the underlying causes of these diseases.

Among the emerging strategies, one promising approach is stem cell therapy.^1^ While the idea of using stem cells to treat neurological disorders is optimistic, launching clinical trials at this stage would be premature. Nevertheless, steady scientific progress continues to strengthen the hope that stem-cell-based therapies could eventually restore and preserve brain and spinal cord function. For each neurological condition, it is now feasible to outline a clear roadmap that identifies the scientific and clinical milestones required before such therapies can reach patients. Before stem-cell treatments are applied in clinical settings, we must achieve precise control over their proliferation and differentiation into specific cell types and ensure that they do not lead to tumor formation. Additionally, their therapeutic effectiveness and mechanisms of action need to be validated in animal models that closely mimic the human disease, both in pathology and symptoms. However, translating results from animals to humans remains challenging due to species-specific differences in neuronal plasticity and limited understanding of disease mechanisms. It is also crucial to learn how to modulate the diseased tissue environment, particularly inflammatory and immune responses, to support effective repair. Ultimately, regardless of the underlying neurobiological promise, the success of stem cell therapies will be judged by their ability to safely deliver long-lasting, meaningful improvements in the quality of life for patients with neurological disorders.

New approaches have recently emerged for converting stem cells into functional neurons using a variety of multifactorial methodologies, especially for human mesenchymal stem cells (hMSCs).^2^ These are multipotent cells and can be differentiated into different types of cells, including neurons. MSCs were isolated from human bone marrow and defined based on their fibroblast-like shape, rapid proliferation rate, and ability to develop into adipocytes, chondrocytes, and osteocytes.^3^ MSCs offer multiple advantages: they are simple to harvest using noninvasive procedures, have a minimal risk of immunological rejection, and do not cause graft-versus-host disease.^1^ In addition to bone marrow, MSCs have been successfully extracted from adipose tissue, synovial fluid, dental pulp, placenta, and even the nasal epithelium, making them extensively available for research and therapy.^2^ Their exceptional adaptability and capacity to preserve stemness even after cryopreservation lend support to their use in clinical settings. Recent research has investigated MSCs’ ability to trans-differentiate into neuronal lineages, suggesting a potential approach for regenerative therapy for neurodegenerative diseases.

Furthermore, MSCs can be differentiated into neuronal cells employing a variety of techniques and inducers, including growth factors, cocultivation with neural lineage cells, chemical substances, gene transfection miRNA, and compounds known as small molecules. One of the most promising directions is to utilize small molecules to influence stem cell differentiation. Furthermore, substances including xanthene derivatives, imidazole-based chemicals, and complex chemical cocktails have been studied for their ability to stimulate neuronal development. Researchers have identified imidazole-based Glycogen synthase kinase-3β (GSK-3β) inhibitors as prospective single-molecule therapeutics capable of stimulating the trans-differentiation of hMSCs into neurons.^1^ GSK-3β regulates several biological processes, including neural growth. Imidazole-based drugs block this enzyme, which not only promotes neuronal development but also has therapeutic promise for neurological diseases. This approach eliminates the problems associated with multidrug “chemical cocktails” and promotes a streamlined, tailored approach to cell-based neurotherapy. These findings are a significant step toward the creation of single molecule neurotherapeutics that combine efficacy, safety, and selectivity for future neural regeneration and brain repair research.

At the same time, fluorescent probes gained attention not only as diagnostic tools but also for real-time observation of neuronal differentiation, owing to their sensitivity, cost-effectiveness, noninvasiveness, and high spatial resolution. NeuO,^4^ a novel fluorescent probe that enables selective staining of live neurons. Unlike traditional methods such as Nissl and Golgi stains, which are limited to fixed tissues, or genetic approaches that can interfere with cellular function, NeuO offers a noninvasive and broadly applicable solution for real-time neuronal imaging. Identified through high-throughput screening of a fluorescence compound library and refined via structure–activity relationship (SAR) studies, NeuO demonstrated high neuron selectivity and compatibility across various species including mice, human stem cell derived neurons, and zebrafish. It effectively stains live neurons without affecting their morphology or electrophysiological properties, and its ability to cross the blood-brain barrier allows for *in vivo* imaging. NeuO is compatible with multiple imaging platforms and cell sorting techniques, making it a valuable tool for neuroscience research focused on neuron development. In addition, multifunctional fluorescent probe, CP-4 ^5^ capable of promoting the trans-differentiation of hMSCs into neurons. Unlike conventional approaches that rely on chemically complex and potentially toxic induction cocktails, CP-4 acts as a single, noncytotoxic molecule that effectively induces neuronal differentiation when combined with fibroblast growth factor (FGF). The probe also displayed strong and stable fluorescence, supporting its utility for extended imaging applications. Although, the dual role of CP-4 as both a diagnostic and therapeutic tool shows promising implications for neuroregenerative therapies, several critical questions remain unanswered. Notably, it is unclear whether the fluorescent probe alone, in the absence of growth factors, possesses any intrinsic ability to induce differentiation. Moreover, even though some probes remain fluorescent for several days postdifferentiation, the relationship between fluorescence intensity and the degree of neuronal differentiation is not explored. Here, we discuss the fluorescence probe with a potential to trans-differentiate the hMSCs to neurons along with the real-time imaging properties (Figure 1).

**Figure 1:**
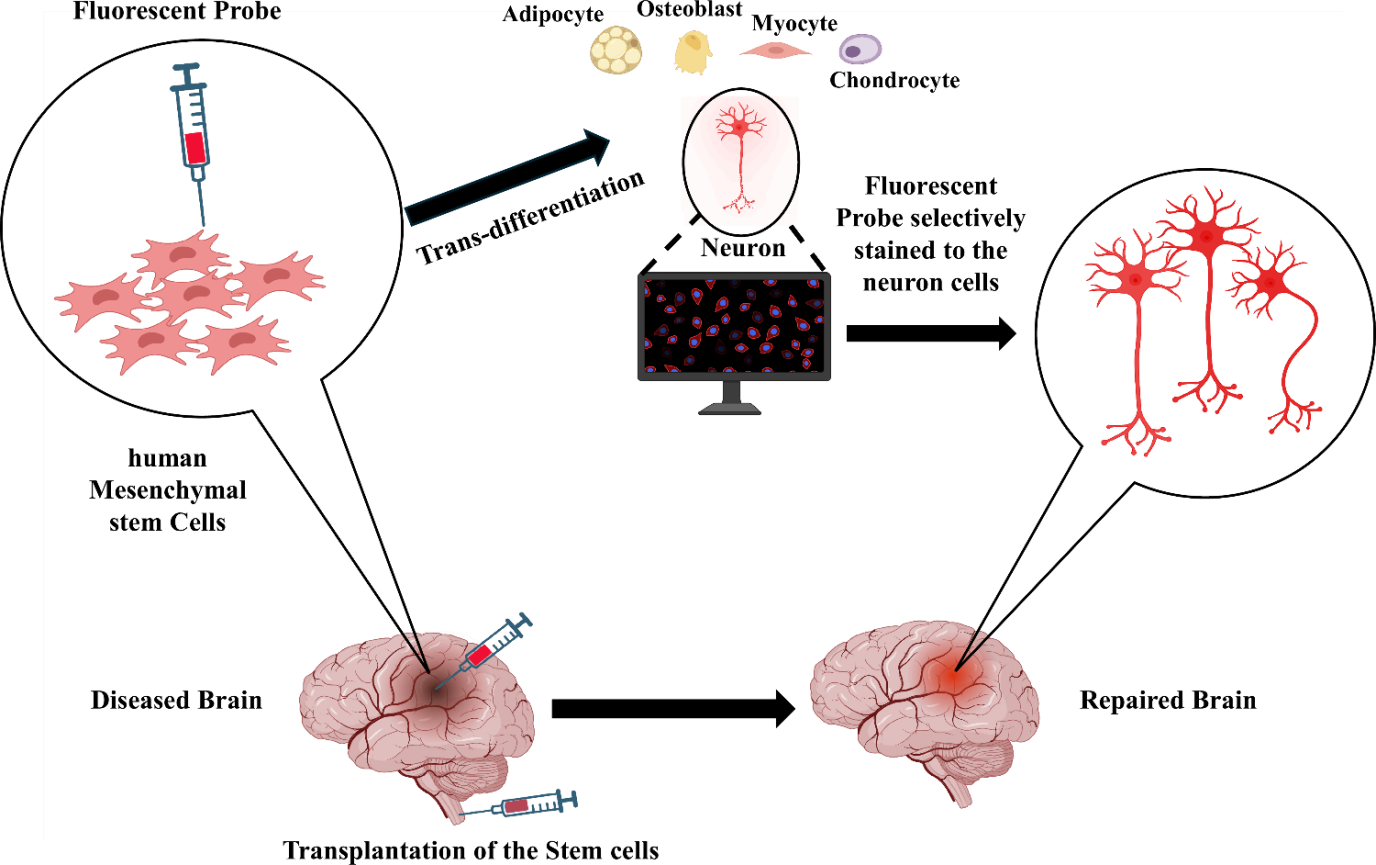
Schematic representation of human Mesenchymal stem cell trans-differentiation into neurons by fluorescent probe to repair the diseased brain. This fluorescent probe has dual role of trans-differentiation to neurons alongside with real time imaging of the neurons.

In summary, most small molecules that promote stem cell differentiation into neurons lack fluorescent properties, while many fluorescent probes require growth factors and do not trigger differentiation on their own. This disconnect highlights a significant gap in the field. Therefore, developing a single fluorescent probe that can both induce neuronal trans differentiation without the need for growth factors and enable real-time imaging would represent a breakthrough in neuroregenerative research. Such dual-function molecules could transform both therapeutic strategies and mechanistic investigations into neurodegenerative diseases.
